# Efficient large amplitude primary resonance in in-extensional nanocapacitors: Nonlinear mean curvature component

**DOI:** 10.1038/s41598-019-56726-y

**Published:** 2019-12-27

**Authors:** Sasan Rahmanian, Shahrokh Hosseini-Hashemi, Masoud SoltanRezaee

**Affiliations:** 1 School of Mechanics Engineering, Iran University of Science and Technology, Narmak, Tehran Iran; 2 Center of Excellence in Railway Transportation, Iran University of Science and Technology, Narmak, 16846-13114 Tehran Iran; 3 Department of Mechanical Engineering, Tarbiat Modares University, Tehran, Iran

**Keywords:** Mechanical engineering, NEMS, Nanosensors, Applied mathematics

## Abstract

In general, the impact of geometric nonlinearity, which arises from geometric relationships governing the motion of constituent particles of elastic mediums, becomes critically important while the system operates under large deformations. In this case, the influence of different physics governing the system dynamics might be coupled with the impact of geometric nonlinearity. Here, for the first time, the non-zero component of the mean curvature tensor is nonlinearly expressed in terms of the middle-axis curvature of a cantilevered beam. To this aim, the concept of local displacement field together with inextensibility condition are employed. A nanowire-based capacitor is assumed to be excited by the electrostatic load that is composed of both DC and AC voltages. The main concern is on the case, in which it is necessary to polarize the electrodes with large amplitude voltages. Other physics, including surface strain energy, size-dependency, and dispersion force are modeled to predict the system response more accurately. Hamilton’s principle is used to establish the motion equation, and the Galerkin method is applied to exploit a set of nonlinear ordinary differential equations (ODEs). Implementing a combination of shooting and arc-length continuation scheme, the frequency and force-displacement behaviors of the capacitor are captured near its primary resonance. The coupled effects of the nonlinear impact factor, surface elasticity and size parameters on the bifurcation point’s loci and dynamic pull-in instability are studied.

## Introduction

Generally speaking, in mechanical elastic structures, the advantage of employing nonlinear dynamics models becomes prominent while the system is assumed to work under large displacements. This is the case that the passive nonlinear terms embedded in system essence are activated. Among the most applicable nowadays engineering systems, nanoelectromechanical systems (NEMS) can be exposed to large amplitude oscillatory while they are actuated with hard excitation, depending on their operating condition to satisfy a desired task. These devices have fixated their position as one of the main components in constructing mass and temperature sensors, actuators, resonators, narrow band filters, energy harvesters, and so on. These devices involve a flexible vibrant core so that their performance depends on the motion of this element. Therefore, it is worthfull to analyze the dynamics of such nanosized structures and find out how their motion characteristics are related to system efficiency.

In nanodevices, the system can experience different types of geometric nonlinearity while they are maintained with large deformation, depending on their configuration. The mid-plane stretching effect and nonlinear curvature are two of the well-known geometric nonlinearities which are, respectively, predominant in fully-clamped and cantilever elastic structures. However, in NEMS devices, there exists other sources of nonlinearity which are due to the presence of displacement-dependent forces such as, electrostatic load and dispersion atomic forces. These are the forces that are inherently introduced by nonlinear formulations in terms of the structures’ displacement and do not matter whether the system is operating under large or small deformations. In the last few decades, a great deal of attention has focused on nonlinear statics^1–11^ and dynamics^12–22^ characteristics of NEMS systems.

Patel *et al*.^23^ proposed a model based on moment-curvature approach to analyze large deflection of nanobeams using the consistent couple stress theory (CCST). They reported on the large elastic deflection of nanobeams subjected to arbitrary inclined terminal load. Chen *et al*.^24^ studied the flapwise vibration of composite laminated Timoshenko nanobeams experiencing rotational movement, considering geometric imperfection. Their model benefited from a re-modified couple stress theory (RMCST) for anisotropic elasticity. The large amplitude free vibration characteristics of nanobeams under electrostatic loading was examined by Wang *et al*.^25^. In their work, both the influences of surface elasticity and Casimir force as a dispersion intermolecular interaction were considered. They also reported on thermal effect which may change the relationship between fundamental natural frequency and applied voltage. Lin *et al*.^26^ examined the influence of Casimir regime on the critical pull-in gap and pull-in voltage of NEMS switches via implementing perturbation analysis. They also investigated the effect of this parameter on the nonlinear behavior of nanoscale electrostatic actuators using a single degree of freedom mass-spring model^27^. Their stability analysis proves that one of the system equilibria is Hopf bifurcation point and the other is unstable saddle-node, while there exists two equilibrium points in the system dynamics. Esfahani *et al*.^28^ exploited the nonlinear frequency-response behavior of electrostatic nanobeam actuator based on nonlocal strain gradient theory. The assessed the roles of different parameters as such, surface elasticity parameter, intermolecular forces and quality factor on the multi-valued zones of the dynamic response. They deduced that surface elasticity induces hardening effect in the nanobeam response, whereas the intermolecular forces come with reverse effect. Dang *et al*.^29^ studied the nonlinear vibration of nanobeams under electrostatic actuation. They employed the equivalent linearization method together with a variational approach to establish a closed-form expression for frequency–amplitude relationship. Ghommem *et al*.^30^ developed a reduced-order model of an electrically-actuated nanocantilever beam having mass at its end to bio-mass sensor applications. They proved that the sensing sensitivity increases for specific mass threshold while reducing the AC load amplitude.

Anderson *et al*.^31^ experimentally proved that the vibration response of beams is remarkably influenced by the impact of geometric nonlinearities, and ignoring these terms results in incorrect outputs. Chaterjee and Pohit^32^ reported on the impact of nonlinear curvature on cantilever beam structures. They examined both the static and dynamic responses of an electrically actuated nanocantilever system, benefiting from Galerkin decomposition method together with a boundary value problem solver. Sheng and Wang^33^ conducted an investigation on parametric excitation of functionally graded (FG) Timoshenko nanobeams under thermal effects. They addressed the impact of length-scale parameter on the one-to-three internal resonance between the first and second transverse natural frequencies of the system. Caruntu *et al*.^34^ conducted an investigation on the parametric resonance voltage response of electrostatically actuated NEMS systems made of cantilever resonators. In their work, the nanobeam was considered to be under pure alternative current (AC) voltage. Since it was assumed that the beam is excited by a soft AC voltage load, the effect of geometric nonlinearity was neglected in their mathematical model. Moreover, they simplified the fractional displacement-dependent force using Taylor series expansion around the zero equilibrium position to make it straightforward for employing multiple time scales method. They also multiplied the both sides of the motion equation by the highest order of the denominator to eliminate the fractional terms rather than directly use the original form of the differential equation while applying the numerical integration method. They studied the Effects of frequency, damping and fringe parameters on the locus of the sub- and super-critical Hopf bifurcations as well as the saddle-node bifurcation points. Dai *et al*.^35^ analyzed the static and dynamic pull-in instabilities of NEM system which is composed of a nanocantilever, as the moveable electrode, based on a nonlinear model. In their work, they equalized the nonlinear inertias, which were originally obtained from the variational approach and appeared as the functional of integral terms, with the nonlinear differential stiffness and velocity-dependent terms. This imposes an artificial hardening effect to the system dynamics. They studied the simultaneous effects of small scale structures and geometric nonlinearity on the pull-in behavior using the modified couple stress theory (MCST). SoltanRezaee *et al*.^3^ presented a nonlinear curvature-based model to analyze the pull-in instability of double-sided nanodevices which can act as variable capacitors. In their model, the electrostatic attractions between the fixed and movable substrates were developed through the piecewise-electrodes. In addition, the influence of surface elasticity, residual surface tension and size parameters on the free vibration characteristics were investigated. Farokhi *et al*.^36^ proposed a size-dependent nonlinear dynamics model for a functionally graded (FG) nanocantilever excited by an external harmonic load. In their model, the nanobeam was not considered to be an element of NEMS device, and therefore its dynamics was not coupled with an electrostatic load. They investigated the influence of size on the system motion employing MCST. They examined the effect of different system parameters, such as the power index of FG material and the impact of small-scale parameter on the system dynamics.

In the present study, the authors aim to investigate the impact of cantilever geometric nonlinearity which is due to large deformation and its coupled effect with the nanosized structures phenomena on the large amplitude resonance characteristics of nanowire-based NEMS systems. For this, it is assumed that the flexible component suffers from large values for the electrostatic voltage load, as hard-type excitation. This is for that the passive nonlinear terms embedded in the system dynamics to be activated, and its consequent impact on the influence of size and surface elasticity parameters becomes prominent. In order to involve the influence of curvatures of the material fibers (mean curvature tensor) into the total potential energy of the flexible component, the modified couple stress theory has been utilized. Actually, this theory accounts for the potential energy which comes from variations in curvatures of the elastic medium fibers after deformation. For nanosized structures, this effect is considerable comparing to that of classical strain energy, and therefore must be included in the model. Herein, the weighted residual method is applied to the original integro-partial differential equation to discretize the motion equation into a set of nonlinear ODEs. Furthermore, the displacement-dependent forces are kept in the fractional form without simplifying them using the Taylor series expansion. The large amplitude softening-type behaviors have been detected in the system response, and the impact of nonlinearity, surface energy, and length-scale parameters on the bending degree of the frequency-response branches are analyzed. Moreover, some types of bifurcation such as saddle-node and Hopf bifurcations as well as dynamic pull-in instability are addressed.

### Nonlinear impact factor

**Figure 1:**
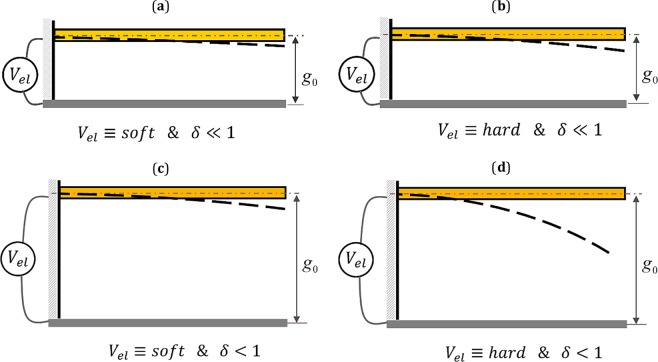
Four different categories concerning on the combined effect of the electrostatic load level and the impact of geometric nonlinearity. Weak nonlinearity and (**a**) soft excitation and (**b**) hard excitation. Strong nonlinearity and (**c**) soft excitation and (**d**) hard excitation.

In cantilever-based NEMS devices, a dimensionless parameter, $\delta ={(\frac{{g}_{0}}{L})}^{2}$, appears in the system equation of motion as the multiplier of nonlinear mass and stiffness matrices, which is the result of geometric nonlinearity assumption. Here, *g*_0_ is the initial gap between the fixed and movable electrodes, and *L* denotes the beam’s length. This is shown in details, in the next section. In the following, this parameter is called as *nonlinear impact factor*. The value of this parameter depends on the value of the initial distance between the two electrodes, for the case that the length of the nanobeam is assumed to be constant. As mentioned previously, the nonlinear electrostatic and intermolecular dispersion forces are independent of that type of nonlinearity coming from the strain-displacement relationships, and can also exist in linear models. In Fig. (1), four different scenarios have been discussed on the interplay between the electrostatic load level and the impact of the parameter, *δ*, that how this parameter can potentially cause the outputs from the linear and nonlinear models to differ.

It is worth mentioning that the system nonlinear model provides us with highly precise results comparing to the linear model, depending on whether the system moves under large deformations or not. In NEMS, the first and necessary condition for which the system is prone to move under large displacement is that the initial gap size to be large enough so that the nanobeam would have permission to greatly be deformed before pull-in instability. However, the second and sufficient condition is, that the system to be actuated as such it displays large amplitude vibration. For the case that the nanobeam is actuated by soft electrostatic voltage amplitude and the gap distance is small, equivalently, the nonlinear impact factor is weak, it is vain to employ the nonlinear mass-stiffness-based model for the NEMS device, see Fig. (1a). Increasing the voltage load up to vicinity of the pull-in voltage as hard excitation, because of the gap smallness between the two conductors, which is equivalent to the case of weak nonlinear impact factor, the system cannot yet experience large amplitude motion. The reason is that the beam does not possess sufficient space to bend in before it crumbles toward the fixed substrate, Fig. (1b). In addition, considering soft excitation for the case that the initial gap is relatively large, the difference between the results obtained from the linear and nonlinear models are negligible while the beam is excited by low-amplitude voltage, as shown in Fig. (1c). Although in this case, the impact of geometric nonlinearity (*δ*) is large (assuming the length of the nanobeam is kept constant), the nonlinear terms are held passive since the beam is not forced to move under large deformation. Actually, the nonlinear model is potentially capable to report different results in comparison with the linear model. If the beam is actuated with larger values of the electrostatic load, it is crucial to benefit from the system nonlinear model in order to accurately predict the NEMS behavior. This is because that the flexible component is assigned to vibrate with large amplitude before dynamic pull-in occurs, Fig. (1d). Since in this work, the main concentration is on investigating the large amplitude oscillations of nanowire-based cantilever capacitor near its primary resonance, we have focused on the conditions depicted in Fig. (1d) when analyzing the effects of nanostructures on the system resonance characteristics.

## Mathematical Modeling

**Figure 2:**
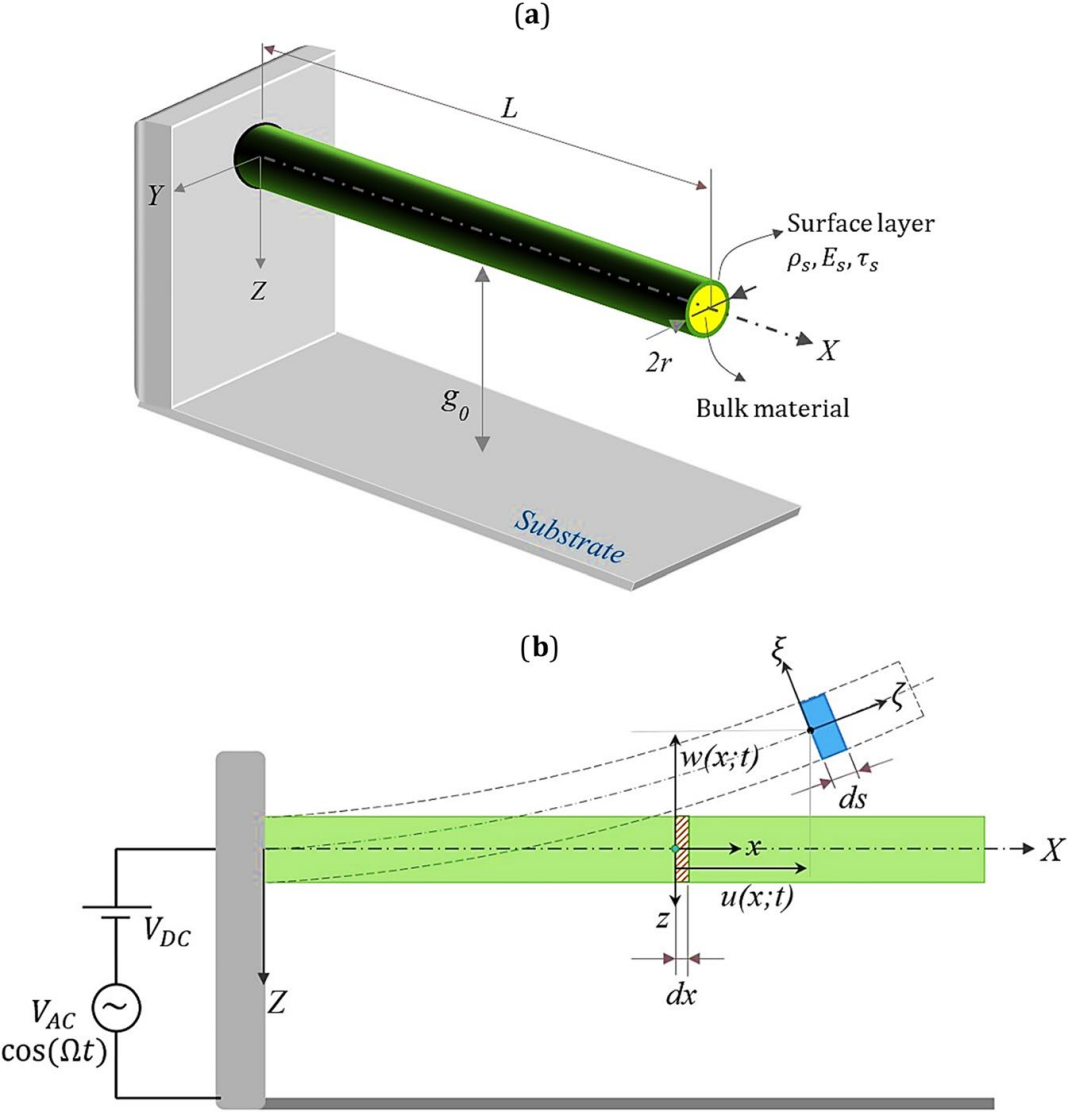
Schematic diagram of a cantilever nanowire under electrostatic actuation.

Figure (2) illustrates a cantilever NEMS device consisting of a movable nanowire and a fixed substrate. The length and the cross-section radius of the nanowire are, respectively, denoted by *L* and *r*, and the initial distance between the two electrodes are represented by *g*_0_. The nanowire is bonded to a surface layer, and it is assumed to be excited by the electrostatic load which is composed of direct current voltage, *V*_*DC*_, and alternative current voltage, *V*_*AC*_. The AC load is applied to the nanowire with the frequency of Ω. As shown in the figure, the *xyz* Cartesian coordinates system is attached to the support at the clamped end of the nanowire and passes through the neutral axis of the undeformed configuration of the beam. The in-plane motion of any material particle located on the middle axis of the beam is defined by the horizontal and vertical displacement components, namely, $u(x;t)$ and $w(x;t)$, respectively.

### Non-conservative work, kinetic and strain energies

The distributed electrostatic load developed between the two electrodes depends on the voltage amplitude applied through the fixed substrate, the initial distance between them and the geometry of the nanodevice. For the case of cylinder-plate capacitor, the electrical force per unit length of the nanowire can be found in the form of^37^,

1 $${F}_{el}=\frac{\pi {({V}_{DC}+{V}_{AC}\cos (\Omega t))}^{2}{\varepsilon }_{0}}{\sqrt{{g}_{0}({g}_{0}+2r)}\,{{\rm{arccosh}}}^{2}(1+\frac{{g}_{0}}{r})}$$

where ${\varepsilon }_{0}=8.854\times {10}^{-12}$ F/m is the permittivity constant of air.

In low nanoscale, two materials separated with narrow distance attract each other through interatomic dispersion forces. In this research, the well-known expressions commonly used to model the Casimir regime for the case of cylinder-plate configuration based on Dirichlet model is considered. Therefore, the Casimir force exerted to unit length of the nanowire can be written as follows^38,39^,

2 $${F}_{cas}=\frac{cP(1+2\,\mathrm{ln}(\frac{{g}_{0}}{r}))}{16\pi {g}_{0}^{3}{\mathrm{ln}}^{2}\,(\frac{{g}_{0}}{r})}$$

where $c=2.998\times {10}^{8}$ m/s is the light speed, and $P=1.05457\times {10}^{-34}$ J.s is the reduced Planck’s constant (Planck’s constant divided by $2\pi$).

It is obvious that, while the nanowire is excited by electrostatic load and commence to vibrate, the distance between the beam element and the stationary ground plate changes to ${g}_{0}-w$. Here, the cross-section radius of the nanowire is considered to be negligible comparing to the initial gap size, namely, ${g}_{0}+r\approx {g}_{0}$. Finally, the non-conservative external force is the summation of electrostatic and Casimir forces and obtained as,

3 $${F}_{ext}={F}_{el}+{F}_{cas}=\frac{\pi {\varepsilon }_{0}{({V}_{DC}+{V}_{AC}\cos (\Omega t))}^{2}}{({g}_{0}-w)\,{{\rm{arccosh}}}^{2}(\frac{{g}_{0}-w}{r})}+\frac{cP(1+2\,\mathrm{ln}(\frac{{g}_{0}-w}{r}))}{16\pi {({g}_{0}-w)}^{3}\,{\mathrm{ln}}^{2}\,(\frac{{g}_{0}-w}{r})}$$

The kinetic energy of the NEMS device consists of two components: one relates to the bulk material, and second is the surface layer contribution, and can be expressed as following,

4 $$T={T}_{B}+{T}_{S}=\frac{1}{2}{\int }_{0}^{L}({(\rho A)}_{eq}{\dot{u}}^{2}+{(\rho A)}_{eq}{\dot{w}}^{2})dx$$

In Eq. (4), overdot indicates partial derivative with respect to time variable, i.e. $\frac{\partial }{\partial t}$, and the following parameters are defined: ${(\rho A)}_{eq}={\rho }_{b}{A}_{b}+{\rho }_{s}{A}_{s}$. ${A}_{b}=\pi {r}^{2}$ is the area of the nanowire cross section, ${A}_{s}=2\pi r$ is the perimeter of the cross section, and the mass density of the bulk and surface layer are denoted by ${\rho }_{b}$ and ${\rho }_{s}$, respectively.

Considering MCST to take into account the effect of nanosized structure, the variational form of the strain energy stored in isotropic linear-elastic materials occupying volume ${\mathcal B}$ is given by,

5 $$\delta {U}_{B}={\int }_{ {\mathcal B} }({\sigma }_{ij}\delta {{\epsilon }}_{ij}+{m}_{ij}\delta {\chi }_{ij})dV$$

where, ${\sigma }_{ij}$, ${{\epsilon }}_{ij}$, ${m}_{ij}$, and ${\chi }_{ij}$ introduce the force-stress tensor, strain tensor, deviatoric part of couple-stress tensor, and symmetric curvature tensor, respectively. Based on MCST, these mechanical quantities can be obtained as^40^,

$${\sigma }_{ij}={C}_{ijkl}{{\epsilon }}_{kl}\,,\,{{\epsilon }}_{ij}=\frac{1}{2}({u}_{i,j}+{u}_{j,i})$$

6 $${m}_{ij}=2\mu {l}^{2}{\chi }_{ij}\,,\,{\chi }_{ij}=\frac{1}{2}({\theta }_{i,j}+{\theta }_{j,i})\,,\,{\theta }_{i}=\frac{1}{2}{\varepsilon }_{ijk}{u}_{k,j}\,;\,i,j=1,2,3$$

where, *μ* is the Lame’s second coefficient, and *l* signifies size-dependency which varies from one material to another or from one scale to another scale, and $\theta$ represents the rotation vector. Also, *C*_*ijkl*_ denotes the elastic constant tensor, and ${\varepsilon }_{ijk}$ is the Cevi-Levita symbol.

Here, it is assumed that the motion of the nanowire element is decomposed into two parts. First, the face of the beam element with the length of *dx*, translates under two rigid body degrees of freedom, $u(x;t)$ and $w(x;t)$, Fig. (2b). Second, the element undergoes deformation using the concept of local displacement field. This is the procedure mentioned in ref. ^41^ to consider the whole motion of beam element traveling from its initial undeformed status to the final deformed configuration. Assuming Euler-Bernoulli beam theory, the local displacement field for any material particles can be written as^41^,

7 $$\begin{array}{c}{u}_{x}(x,y,z;t)={u}_{x}^{0}(x;t)+zsin\Theta (x;t),\,{u}_{y}(x,y,z;t)=0\\ {u}_{z}(x,y,z;t)={u}_{z}^{0}(x;t)-z(1-\,\cos \,\Theta (x;t))\end{array}$$

where,

8 $${u}_{i}=0,\,{u}_{i}^{0}=0\,for\,i=x,y,z;\,\Theta =0,\,e(x;t)=\frac{\partial {u}_{x}^{0}}{\partial x},\zeta (x;t)=\frac{\partial \Theta }{\partial x}$$

here, $\Theta$ is the local rotation of the face of the beam element which is due to bending distortion. $e(x;t)$ is the longitudinal strain of the fiber *dx*, which is extruded from a point locating on the centroidal axis of the beam and is at the distance, *x*, from the left end. $\zeta (x;t)$ is the nonlinear curvature of this fiber after deformation. These two kinematic functions are defined as following,

9 $$e(x;t)=\frac{ds-dx}{dx}={({(1+\frac{\partial u}{\partial x})}^{2}+{(\frac{\partial w}{\partial x})}^{2})}^{0.5}-1$$

10 $$\zeta (x;t)=\frac{(1+\frac{\partial u}{\partial x})\frac{{\partial }^{2}w}{\partial {x}^{2}}-\frac{{\partial }^{2}u}{\partial {x}^{2}}\,\frac{\partial w}{\partial x}}{{({(1+\frac{\partial u}{\partial x})}^{2}+{(\frac{\partial w}{\partial x})}^{2})}^{1.5}}$$

It should be noted that, the length of the middle axis does not experience changes as the nanowire is free from one end. Therefore, the strain component at the neutral axis, $e(x;t)$, is set to be zero for the cantilever boundary conditions, $(e=0)$. Hence, Eq. (9) yields the inextensibility condition as,

11 $${({(1+\frac{\partial u}{\partial x})}^{2}+{(\frac{\partial w}{\partial x})}^{2})}^{0.5}=1$$

Simplifying Eq. (11) using Taylor series expansion and omitting the higher order terms result in,

12 $$\frac{\partial u(x;t)}{\partial x}\approx -\,\frac{1}{2}{(\frac{\partial w(x;t)}{\partial x})}^{2}$$

Introducing Eqss. (11) and (12) into Eq. (10), the nonlinear curvature can be expressed only in terms of the transverse displacement.

13 $$\zeta (x;t)={w}_{xx}+\frac{1}{2}{w}_{xx}{w}_{x}^{2}$$

Implementing Eqs. (6)–(8), the nonzero components of the strain tensor, mean curvature tensor, stress tensor, and couple-stress tensor are obtained as follows,

14 $$\begin{array}{c}{{\epsilon }}_{xx}(x,z;t)=e(x;t)-z\zeta (x;t),\,{\chi }_{xy}(x,z;t)={\chi }_{yx}=\frac{1}{4}(z{\zeta }^{2}+\zeta )\\ {\sigma }_{xx}(x,z;t)=E{{\epsilon }}_{xx}(x,z;t),\,{m}_{xy}={m}_{yx}=\frac{\mu {l}^{2}}{2}(z{\zeta }^{2}+\zeta )\end{array}$$

Substituting Eq. (13) and (14) into Eq. (5), and applying $e=0$, the strain energy stored in the bulk is obtained as,

15 $$\delta {U}_{B}={\int }_{0}^{L}({E}_{b}{I}_{b}({w}_{xx}+\frac{1}{2}{w}_{xx}{w}_{x}^{2})+\frac{\mu {l}^{2}{A}_{b}}{4}[\frac{{r}^{2}}{2}{({w}_{xx}+\frac{1}{2}{w}_{xx}{w}_{x}^{2})}^{3}+({w}_{xx}+\frac{1}{2}{w}_{xx}{w}_{x}^{2})])\delta ({w}_{xx}+\frac{1}{2}{w}_{xx}{w}_{x}^{2})dx$$

For the surface layer surrounding the bulk material, the stress induced in the perimeter surface of the wire can be given by^42^,

16 $${\tau }_{xx}(x,z;t)={\tau }_{0}+{E}_{s}{{\epsilon }}_{xx}(x,z;t)$$

where, ${\tau }_{0}$ and ${E}_{s}$ are the residual surface stress, and the surface Young’s modulus, respectively. The variational form of the surface strain energy due to bending of the structure can be written in the following form

17 $$\delta {U}_{S}^{1}=\mathop{\oint }\limits_{\partial S}{\tau }_{xx}\delta {{\epsilon }}_{xx}dS={E}_{s}{I}_{s}{\int }_{0}^{L}\zeta (x,z;t)\delta \zeta dx;\,{I}_{s}=\pi {r}^{3}$$

On the other hand, when the beam curvature is nonzero, because of the initial surface energy which is due to residual surface stress, a distributed transverse load, $q(x;t)$, is applied to the surface^42^

18 $$q(x;t)=4{\tau }_{0}r\zeta (x;t)$$

Thus, the second part of the surface strain energy corresponding to the load mentioned by Eq. (18) can be written as

19 $$\delta {U}_{S}^{2}={\int }_{0}^{L}q(x;t)\delta wdx$$

### Motion equation

In this section, the extended Hamilton’s principle is employed in order to establish the partial differential equation of motion governing the NEMS dynamics. This implies that during a particle’s motion, its total amount of energy is held at minimum level keeping the equilibrium.

20 $${\int }_{{t}_{1}}^{{t}_{2}}(\delta T-(\delta {U}_{B}+\delta {U}_{S}^{1}+\delta {U}_{S}^{2})+{\int }_{0}^{L}({F}_{el}+{F}_{cas})\delta wdx)dt=0$$

By substituting the expression obtained for the kinetic and strain energies as well as the work done by the external forces into Eq. (20), and after some straightforward mathematical manipulation, and also, considering the influence of viscous damping *C*_*d*_, the nonlinear motion equation associated to the transverse vibration of the nanowire is obtained as,

21 $$\begin{array}{c}{(\rho A)}_{eq}(\ddot{w}+{w}_{x}{\int }_{0}^{x}({\ddot{w}}_{x}{w}_{x}+{\dot{w}}_{x}^{2})dx+{w}_{xx}{\int }_{L}^{x}({\int }_{0}^{x}({\ddot{w}}_{x}{w}_{x}+{\dot{w}}_{x}^{2})dx)dx)+{C}_{d}\dot{w}\\ \,+\,{(EI)}_{eq}[{w}_{xxxx}+{w}_{xxxx}{w}_{x}^{2}+4{w}_{xxx}{w}_{xx}{w}_{x}+{w}_{xx}^{3}]+\frac{\mu {A}_{b}{r}^{2}{l}^{2}}{4}\frac{{\partial }^{2}({w}_{xx}^{3})}{\partial {x}^{2}}\\ \,+\,\frac{\mu {A}_{b}{l}^{2}}{4}[{w}_{xxxx}+\frac{{\partial }^{2}({w}_{xx}{w}_{x}^{2})}{\partial {x}^{2}}-\frac{\partial ({w}_{xx}^{2}{w}_{x})}{\partial x}]\\ \,-4{\tau }_{0}r\,{w}_{xx}(1+\frac{1}{2}{w}_{x}^{2})=\frac{\pi {\varepsilon }_{0}{({V}_{DC}+{V}_{AC}\cos (\Omega t))}^{2}}{({g}_{0}-w{){\rm{arccosh}}}^{2}(\frac{{g}_{0}-w}{r})}\\ \,+\,\frac{cP(1+2\,\mathrm{ln}(\frac{{g}_{0}-w}{r}))}{16\pi {({g}_{0}-w)}^{3}\,{\mathrm{ln}}^{2}(\frac{{g}_{0}-w}{r})}\end{array}$$

Subject to the following boundary conditions,

22 $$w(0;t)={w}_{x}(0;t)=0;\,{w}_{xx}(L;t)={w}_{xxx}(L;t)=0$$

here, (·)_*x*_ stands for partial derivatives with respect to axial coordinate. ${(EI)}_{eq}={E}_{b}{I}_{b}+{E}_{s}{I}_{s}\cdot {E}_{b}$ and ${I}_{b}$ represent the Young’s modulus of the bulk and the second moment of area of the beam cross section, respectively.

In order to simply carry out a parametric investigation, the dimensionless form of the motion equation is obtained through introducing the following non-dimensional parameters,

23 $$\tilde{w}=\frac{w}{{g}_{0}},\,\tilde{x}=\frac{x}{L},\,\tilde{t}=\frac{t}{{t}^{\ast }},\,\tilde{\Omega }=\Omega {t}^{\ast }$$

where, ${t}^{\ast }$ is defined as a characteristic time (timescale), and given

24 $${t}^{\ast }=\sqrt{\frac{{(\rho A)}_{eq}{L}^{4}}{{(EI)}_{eq}}}$$

Substituting Eqs. (23) and (24) into Eqs. (21) and (22), and removing the tildes with the aim of simplicity, the dimensionless motion’s equation and the corresponding boundary conditions are obtained:

25 $$\begin{array}{c}\ddot{w}+\delta {w}_{x}{\int }_{0}^{x}({\ddot{w}}_{x}{w}_{x}+{\dot{w}}_{x}^{2})dx+\delta {w}_{xx}{\int }_{1}^{x}({\int }_{0}^{x}({\ddot{w}}_{x}{w}_{x}+{\dot{w}}_{x}^{2})dx)dx+{\alpha }_{4}\dot{w}\\ \,+\,{w}_{xxxx}(1+\delta {w}_{x}^{2})+\delta (4{w}_{xxx}{w}_{xx}{w}_{x}+{w}_{xx}^{3})+\delta {\alpha }_{2}\frac{{\partial }^{2}({w}_{xx}^{3})}{\partial {x}^{2}}\\ \,+\,{\alpha }_{3}[{w}_{xxxx}+\delta \frac{{\partial }^{2}({w}_{xx}{w}_{x}^{2})}{\partial {x}^{2}}-\delta \frac{\partial ({w}_{xx}^{2}{w}_{x})}{\partial x}]+{\alpha }_{5}\,{w}_{xx}(1+\frac{1}{2}\delta {w}_{x}^{2})\\ \,=\,\frac{{\alpha }_{6}{(1+R\cos (\Omega t))}^{2}}{(1-w){{\rm{arccosh}}}^{2}({b}_{0}(1-w))}+\frac{{\alpha }_{7}(1+2\,\mathrm{ln}\,({b}_{0}(1-w)))}{{(1-w)}^{3}{\mathrm{ln}}^{2}({b}_{0}(1-w))}\end{array}$$

26 $$w(0;t)={w}_{x}(0;t)=0;\,{w}_{xx}(1;t)={w}_{xxx}(1;t)=0$$

The non-dimensional coefficients appearing in Eq. (25) are introduced as,

27 $$\begin{array}{c}\delta ={(\frac{{g}_{0}}{L})}^{2},\,R=\frac{{V}_{AC}^{2}}{{V}_{DC}^{2}},\,{\alpha }_{2}=\frac{\mu {A}_{b}{r}^{2}{l}^{2}}{4{L}^{2}{(EI)}_{eq}},\,{\alpha }_{3}=\frac{\mu {A}_{b}{l}^{2}}{4{(EI)}_{eq}},\,{\alpha }_{4}=\frac{{c}_{d}{L}^{2}}{\sqrt{{(\rho A)}_{eq}{(EI)}_{eq}}},\,{\alpha }_{5}\\ =-\frac{4{\tau }_{0}r{L}^{2}}{{(EI)}_{eq}}\,{\alpha }_{6}=\frac{\pi {\varepsilon }_{0}{V}_{DC}^{2}{L}^{4}}{{g}_{0}^{2}{(EI)}_{eq}},\,{\alpha }_{7}=\frac{cP{L}^{4}}{16\pi {g}_{0}^{4}{(EI)}_{eq}}\end{array}$$

## Reduced-Order Model of the NEMS

In this study, Galerkin decomposition method has been employed to separate the spatial and time variables, and convert the nonlinear partial differential equation into *N*-second order nonlinear ODEs. Then, the system dynamics is described by the so-called reduced-order model (ROM). In this approach, it is assumed that the displacement function is a linear combination of the eigenfunctions of the corresponding linear undamped system, ${\varphi }_{i}(x)$, given

28 $$w(x;t)=\mathop{\sum }\limits_{i=1,2,\ldots }^{N}{\varphi }_{i}(x){q}_{i}(t)$$

where, ${q}_{i}(t)$ indicates the ${i}^{th}$ time-dependent generalized coordinate. Moreover, ${\varphi }_{i}$’s are normalized such that ${\int }_{0}^{1}{\varphi }_{i}{\varphi }_{j}dx={\delta }_{ij}$, and are the solution to the following eigenvalue problem,

29 $$(1+{\alpha }_{3}){\varphi }_{i}^{\text{'}\text{'}\text{'}\text{'}}+{\alpha }_{5}{\varphi ^{\prime\prime} }_{i}={\omega }_{i}^{2}{\varphi }_{i}\,;{\varphi }_{i}(0)={\varphi ^{\prime} }_{i}(0)=0,\,{\varphi ^{\prime\prime} }_{i}(1)={\varphi }_{i}^{\text{'}\text{'}\text{'}}(1)=0$$

where, ${\omega }_{i}$ is the ${i}^{th}$ dimensionless natural frequency of the corresponding linear system. It is worth mentioning that, the effects of size and surface parameters are included in the linear mode shape and natural frequency. By substituting Eq. (28) into Eq. (25), and using Eq. (29) to eliminate ${\varphi }_{i}^{\text{'}\text{'}\text{'}\text{'}}$, and multiplying both sides by ${\varphi }_{n}$, by means of Galerkin technique, and ultimately integrating the obtained result over the interval of [0, 1], reduces to a set of nonlinear ODEs in terms of the generalized coordinates, ${q}_{i}$. The vectorial form of the system equations of motion can be written as,

30 $$\begin{array}{c}([{\bf{I}}]+\delta [{{\bf{M}}}_{{\rm{nl}}}^{1}({\bf{q}})]+\delta [{{\bf{M}}}_{{\rm{nl}}}^{3}({\bf{q}})])\{\ddot{{\bf{q}}}\}+{\alpha }_{4}\{\dot{{\bf{q}}}\}\\ +\,(\begin{array}{c}[{\omega }_{n}^{2}]+\delta [{{\bf{M}}}_{{\rm{nl}}}^{2}({\bf{q}})]+\delta [{{\bf{M}}}_{{\rm{nl}}}^{4}({\bf{q}})]+\\ \delta [{{\bf{K}}}_{{\rm{nl}}}^{1}({\bf{q}})]+4\delta (1+{\alpha }_{3})[{{\bf{K}}}_{{\rm{nl}}}^{2}({\bf{q}})]+\\ \delta (1+{\alpha }_{3})[{{\bf{K}}}_{{\rm{nl}}}^{3}({\bf{q}})]+\frac{1}{2}\delta {\alpha }_{5}[{{\bf{K}}}_{{\rm{nl}}}^{4}({\bf{q}})]+\\ \frac{3\delta {\alpha }_{2}}{1+{\alpha }_{3}}[{{\bf{K}}}_{{\rm{nl}}}^{5}({\bf{q}})]+\frac{6\delta {\alpha }_{2}}{1+{\alpha }_{3}}[{{\bf{K}}}_{{\rm{nl}}}^{6}({\bf{q}})]\end{array})\{{\bf{q}}\}={\alpha }_{6}\{{{\bf{F}}}_{{\rm{el}}}({\bf{q}})\}+{\alpha }_{7}\{{{\bf{F}}}_{{\rm{cas}}}({\bf{q}})\}\end{array}$$

here, $[{\bf{I}}]$ is the identity matrix, and $[{{\bf{M}}}_{{\rm{nl}}}^{{\rm{i}}}]$ and $[{{\bf{K}}}_{{\rm{nl}}}^{{\rm{i}}}]$ are, respectively, the nonlinear mass and stiffness matrices so that their components as well as those of electrostatic and Casimir forces are presented in Appendix. (A). As observed in Eq. (30), the nonlinear impact factor, i.e. $\delta$, is multiplied to all the nonlinear matrices. Setting this parameter equal to zero, independent to the gap size, yields the linear model in which the effects of nonlinear inertia and curvature are neglected.

The electrostatic and Casimir forces are held in their original fractional form that imposes extreme nonlinearities to the system dynamics. Although this requires high computational cost while these two terms are considered intact, the system response can be estimated with high precision. In the rest of the paper, first, the effect of mode numbers on the system response convergence is studied, and then, the influences of the nonlinear impact factor, small-scale and surface elasticity parameters on the frequency- and force-amplitude response of the NEMS device in the vicinity of the primary resonance are investigated under hard voltage loads.

## Results and Discussion

In this section, the numerical simulation based on the combined shooting-arclength continuation method has been schemed to capture the steady-state response of the system. In order not to increase the text content, the details of the employed numerical procedure are ignored to be mentioned here. The displacement amplitude of the wire tip is considered as the system response. The same nanowire examined in ref. ^10^ is selected as a case study. The geometrical properties of the NEMS system together with the mechanical characteristics related to the bulk and surface layer are listed in Table 1.

**Table 1:** Mechanical properties and the geometrical dimensions of the NEMS device.

Parameter	Elastic Young’s modulus (*E*)	Surface elasticity (*E*_*S*_)	Poisson ratio (*v*)	Residual surface stress $({\tau }_{0})$	wire radius (*r*)	wire length (*L*)	Initial gap (*g*_0_)
value	68.5 GPa	6.09 N/m	0.33	0.91 N/m	2 nm	50 nm	35 nm

It should be noted that the initial gap value varies while studying the impact of geometric nonlinearity on the system dynamics. For the remaining analysis, the initial distance between the two conductors is considered as the same value mentioned in Table 1.

**Figure 3:**
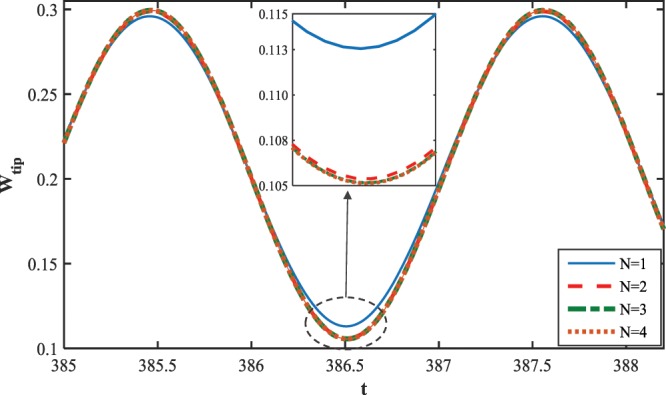
The influence of the mode number on the system response convergence.

As seen in the previous section, the wire displacement was expanded in terms of the linear eigenfunctions, which are known as comparison functions for the nonlinear system. First, the sufficient number of modes used in the series must be evaluated to endure solution convergence and achievement to a satisfactory level of accuracy. Hence, the influence of mode number employed in Galerkin method is examined on the time-history response of the system, Fig. (3). As observed in the figure, the solution path obtained for the case of $N=3$ coincides the system response corresponding to the case of $N=4$. Therefore, to decrease the computational cost keeping the adequate accuracy, a three-mode approximation is considered to report on the dynamics characteristics of the NEMS system. To this aim, the three second-order ODEs are transferred to a system of first-order nonlinear differential equations in six-dimension state space. Here, the couple-stress parameter is assumed to be $l=\frac{r}{6}$.

**Figure 4:**
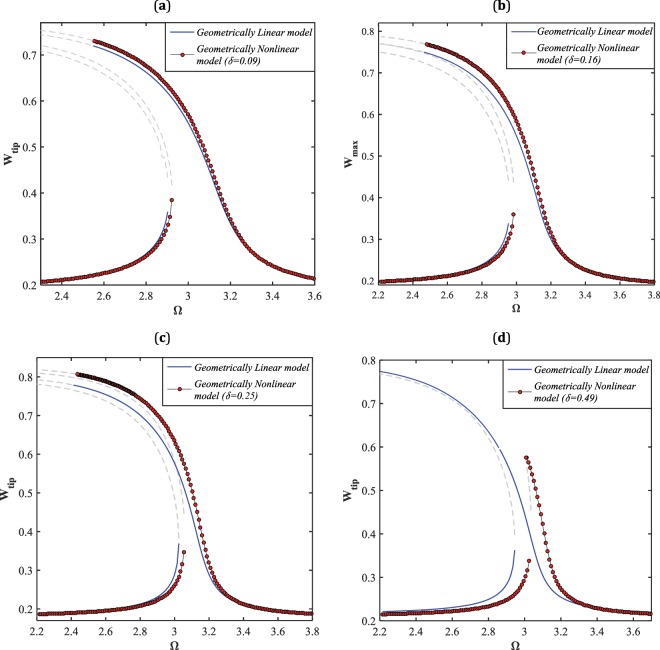
The frequency-displacement curves. The effect of the nonlinear impact factor on the system response near its primary resonance, for ${V}_{AC}=0.1\,V$ and (**a**) ${V}_{DC}=3\,V$, (**b**) ${V}_{DC}=4.5\,V$, (**c**) ${V}_{DC}=6\,V$, and (**d**) ${V}_{DC}=10\,V$.

**Figure 5:**
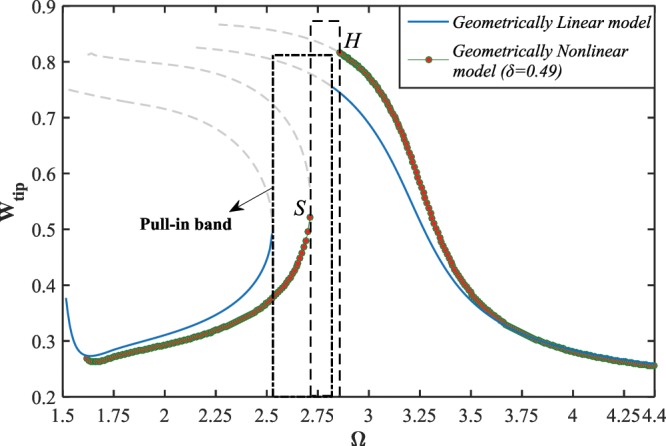
The difference between the linear and nonlinear models on dynamic pull-in behavior, for ${V}_{DC}=10\,V,{V}_{AC}=1\,V,\,{\rm{and}}\,\delta =0.49$.

**Figure 6:**
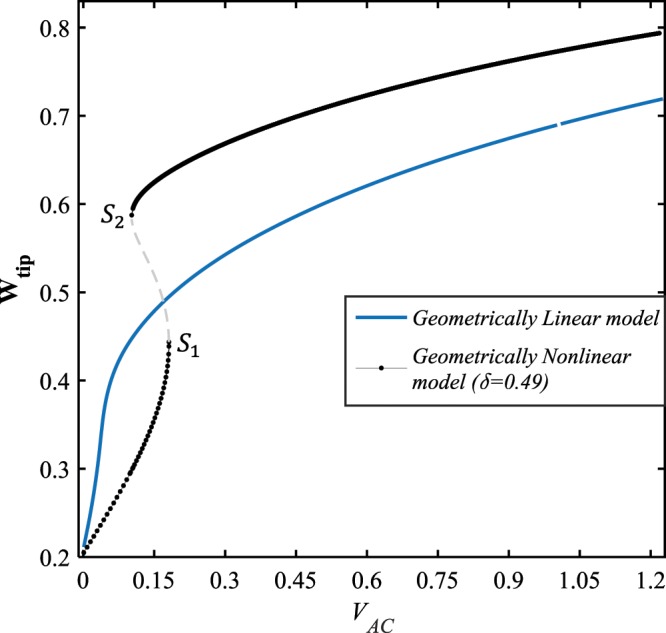
The force-response behavior of the system; the linear and nonlinear models, for ${V}_{DC}=10\,V,\,\Omega =3\,{\rm{and}}\,\delta =0.49$.

Figure (4) depicts the influence of the nonlinear impact factor $(\delta )$, appearing from the nonlinear geometric relationships, on the frequency-response behavior of the NEMS device near the primary resonance, for the case of hard excitation, ${V}_{DC}=10\,V$ and ${V}_{AC}=0.1\,V$. It is seen that for lower impacts of nonlinearity, $\delta =0.09$ for instance, there exists a slight deviation between the results obtained from linear and nonlinear models, Fig. (1a). In this case, the stable and unstable solution branches are relatively far from each other, and the resonance region is broad band. Increasing the impact of geometric nonlinearity, namely, increasing the initial gap value, the deviation between the large-amplitude stable solutions corresponding to the linear and nonlinear models as well as the unstable branches enhances. Moreover, the resonance zone shrinks and the horizontal distance between the two stable solutions decreases while the impact of nonlinearity is intensified, as seen in Fig. (1c). For the case that the impact of nonlinearity reaches nearly one half, it is observed that the linear model predicts an extreme softening-type behavior with large displacement amplitude, whereas the nonlinear model intensively reduces the softening degree of the system response as well as the maximum amplitude. Furthermore, there occurs a right-shift to the resonance region reported by the nonlinear model while the parameter $\delta$ grows. It can be concluded that, for low values of the nonlinear impact factor, it is safe to implement the linear model instead of the nonlinear model even the nanowire is under hard excitation. However, for higher values of the parameter, it is vital to employ the nonlinear model while the nanostructure is actuated by hard-amplitude electrostatic voltages. It is worth mentioning that for the analyses performed through Figs. (4–6), the effects of size and surface elasticity are ignored, and only the impact of geometric nonlinearity on the system dynamics characteristics has been assessed.

The dynamic pull-in instability behavior of the NEMS system is studied under both the linear and nonlinear models, as shown in Fig. (5). As illustrated in the figure, while the parameter $\delta$ is set to be zero in Eq. (30), the pull-in band covers a wider range of the excitation frequency comparing to what is reported by applying the nonlinear model. Moreover, the range of the frequency in which the nanowire becomes dynamically unstable shifts to the higher frequencies as the influence of the geometric nonlinearity is considered in the mathematical model. The large-amplitude solution manifold obtained from the nonlinear model overestimates that value calculated from the linear model, however, this scenario is inversed about the left and low-amplitude branches. In addition, the saddle-node and Hopf bifurcations occur, respectively, at points *S* and *H*.

Figure (6) shows the force-amplitude response of the system for two cases, the geometrically linear and nonlinear models. Here, the bias DC voltage load is set to be ${V}_{DC}=10\,V$, and it is assumed that the AC load amplitude is swept from 0 up to 1 volt. As seen in the figure, for the nonlinear model $(\delta =0.49)$, the system response contains two stable and one unstable solution manifolds which are connected through two saddle-node bifurcation points, whereas no bifurcation occurs while the impact of nonlinearity is ignored. Furthermore, it is observed that for lower values of the AC voltage load, the linear model predicts the system response with larger amplitude, comparing to the nonlinear model. Contrarily, as the external excitation becomes harder, the system response assessed by the nonlinear model possesses greater amplitudes than what is estimated by the geometrically linear model assumption.

**Figure 7:**
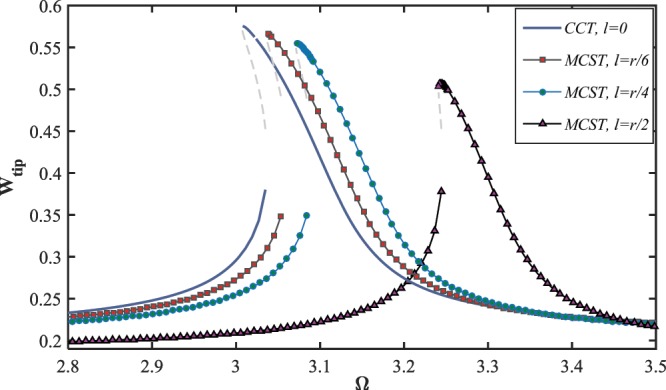
The effect of size-dependency on the frequency-response behavior of the NEMS, for ${V}_{DC}=10\,V,\,{V}_{AC}=0.1\,V\,{\rm{and}}\,\delta =0.49$.

Hereinafter, the nonlinear model, including both the effects of nonlinear inertia and curvature, is utilized to carry out the following analysis on the nanostructure effects on the system dynamics. The influence of the length-scale (or couple stress parameter) on the large amplitude resonant response of the NEMS system is demonstrated in Fig. (7). It can be seen that the maximum amplitude of the tip displacement is remarkably affected by the size parameter, so that enhancing the impact of couple-stress parameter causes the nanostructure becomes stiffer, yielding reduction in the response level. In addition, it interacts with the softening effect imposed by the electrostatic load, and consequently diminishes the degree of the softening-type behavior. Furthermore, the range of the excitation frequency in which the nanowire undergoes resonant retards to the higher frequencies.

**Figure 8:**
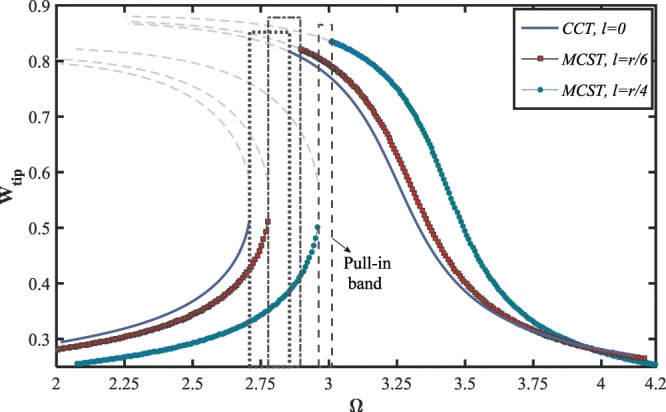
The effect of size-dependency on dynamic pull-in instability of the NEMS, for ${V}_{DC}=10\,V,\,{V}_{AC}=1\,V,\,{\rm{and}}\,\delta =0.49$.

Figure (8) illustrates the frequency-displacement behavior of the system, addressing the dynamic pull-in instability of the nanowire under the influence of the couple-stress parameter. As shown, the curves contain one Hopf and one saddle-node bifurcation points for all cases. The width of the pull-in band reduces while the couple-stress contribution is considered in the strain energy stored in the bending nanowire. The amplitude of the left and right stable solution manifolds, respectively, decreases and increases as the intensity of size-dependency is strengthened. As previous, the resonance region moves to the larger excitation frequencies while the value of the small-scale parameter enhances.

**Figure 9:**
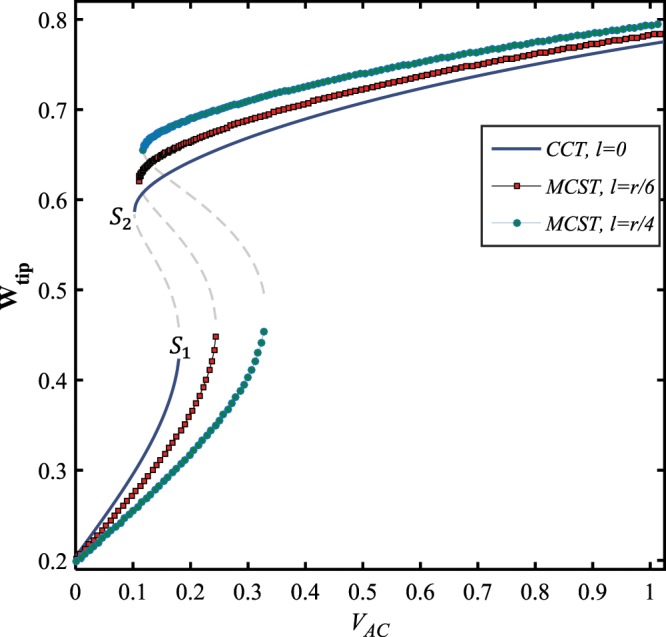
The effect of size-dependency on the force-response behavior of the system, for ${V}_{DC}=10\,V,\,\Omega =3\,{\rm{and}}\,\delta =0.49$.

The influence of the small-scale parameter, which signifies the curvature of the fibers at any material points of the structure, on the force-displacement behavior of the nanoelectromechanical system is depicted in Fig. (9). It is seen that the two stable solution manifolds bifurcate at two saddle-node bifurcation points, *S*_1_ and *S*_2_, so that the first bifurcation point loci shifts to the right at about a certain value of the tip deflection, ${{\bf{W}}}_{{\bf{t}}{\bf{i}}{\bf{p}}}\approx 0.45$; whereas the location of the second saddle-node moves upward to higher values of the tip displacement around a specific AC voltage value, ${V}_{AC}\approx 0.11\,V$. In addition, while the impact of size-dependency enhances, the amplitude of the upper stable branch increases, and the lower stable branch is stretched to the right covering the wider range of AC load.

**Figure 10:**
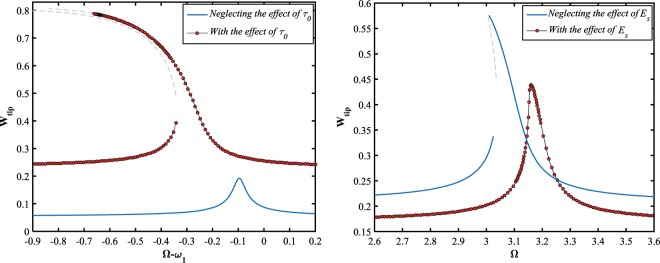
The influence of the surface elasticity parameters; (**a**) residual surface stress for ${V}_{DC}=5.5\,V$, and (**b**) surface elastic modulus for ${V}_{DC}=10\,V$, on the frequency-response behavior of the NEMS. ${V}_{AC}=0.1\,V\,{\rm{and}}\,\delta =0.49$.

Here, the steady-state characteristics of the system dynamics are investigated with and without considering the surface energy contribution in the total strain energy. Figure (10a) shows that the following results are reported while the effect of residual surface stress is included in the model. First, the solution level significantly grows, second, the two unstable solution manifolds intersect the stable branches at the saddle-node and hopf bifurcation points, appearing in the frequency-response behavior of the resonator, and third, the large-amplitude solution branches extremely bend to the left nominating the softening-type behavior. However, the surface elasticity modulus has an inverse effect on the NEMS dynamics comparing to the residual surface stress. It is observed that the unstable manifold disappears and the maximum tip amplitude is suppressed down to about its one half, and furthermore, the branches tend to become vertically straight in the neighbor of the resonance frequency, as the system dynamics is captured in the presence of the surface elasticity modulus, Fig. (10b).

**Figure 11:**
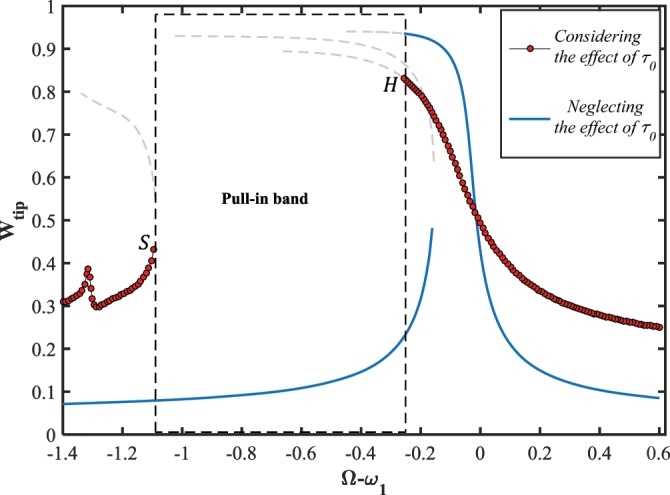
The influence of residual surface stress on the frequency-response behavior of the system, for ${V}_{DC}=5\,V,\,{V}_{AC}=1\,V,\,{\rm{and}}\,\delta =0.49$.

As the other crucial result that can be addressed here, is about the influence of the residual surface stress, ${\tau }_{0}$, on the pull-in instability behavior of the system. To this purpose, it is supposed that the electrostatic voltages amplitude are tuned to be as, ${V}_{DC}=5\,V$ and ${V}_{AC}=1\,V$. As observed in Fig. (11), the manifolds of the frequency-displacement curve are close to each other, and the resonant behavior takes place in a narrow band of excitation frequency while the impact of the parameter is ignored. However, regarding the initial surface tension causes that the stable solution branches take far away and the nanowire crumbles toward the stationary electrode (dynamic pull-in) in a wide range of the stimulation frequency. In other words, for the case of hard excitation, the numerical simulation predicts artificial stable limit cycles near the first-mode primary resonance while the influence of the residual surface stress is not embedded in the system dynamics.

**Figure 12:**
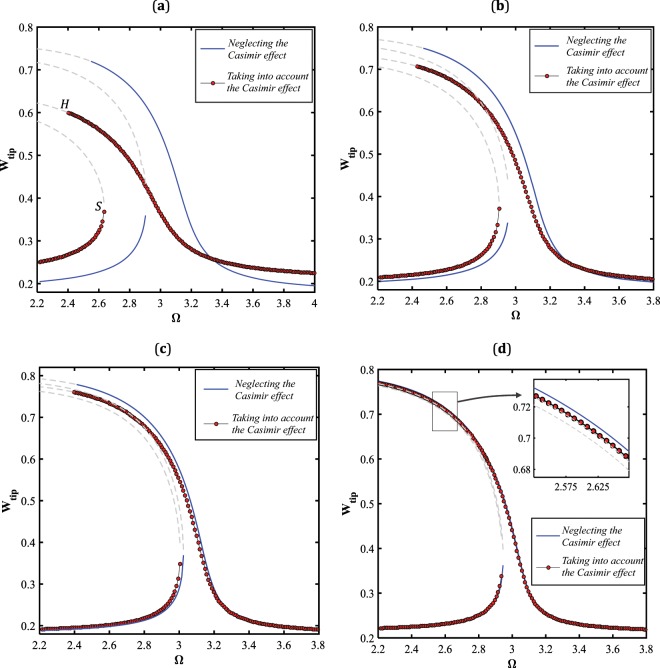
The influence of Casimir regime on the frequency-response behavior of the system, for ${V}_{AC}=0.1\,V$ and (**a**) ${g}_{0}=15\,nm\,{\rm{and}}\,{V}_{DC}=3\,V$, (**b**) ${g}_{0}=20\,nm\,{\rm{and}}\,{V}_{DC}=4.5\,V$, (**c**) ${g}_{0}=25\,nm\,{\rm{and}}\,{V}_{DC}=6\,V$, and (**d**) ${g}_{0}=35\,nm\,{\rm{and}}\,{V}_{DC}=10\,V$.

As the initial gap size is supposed to vary in the range of 15 *nm*~35 *nm*, the Casimir intermolecular dispersion force becomes dominant comparing to van der Waals regime^25,43,44^, and therefore in the following, the impact of this interatomic force developed between the capacitor elements are examined. The effect of the dispersion interatomic regime counted as another source of nonlinearity, which is independent of geometric nonlinearity, is studied on the nature of the system periodic orbits. Here, four different values for the initial gap is considered, and the amplitude of the system response is captured in the presence and absence of the Casimir force. It is illustrated that the deviation between the solutions obtained with and without considering Casimir force is intensively apparent, for the case of narrower gap, Fig. (12a). It is seen in the figure, that the amplitude of the stable periodic solutions away from the resonance region increase, whereas the maximum amplitude (occurs at the Hopf bifurcation point) decreases while the system dynamics is simulated under the influence of the interatomic force. Comparing the curves plotted in Fig. (12a), reveals that the presence of the Casimir force lead to that the large-amplitude manifold bends more to the left, imposing an extreme softening behavior; and also, the nanowire oscillates in a broader band of resonance frequency. As the initial gap value increases, the dependency of the system response to the interatomic attraction is weakened, Fig. (12b,c). For the gap size of ${g}_{0}=35\,nm$, the solution branches corresponding to the two cases, i.e. with and without considering the Casimir effect, approximately coincide, Fig. (12d). Since the nonlinear dispersion force highly affects the system motion for lower gap sizes, the influence of this physics on pull-in instability is examined in the following.

**Figure 13:**
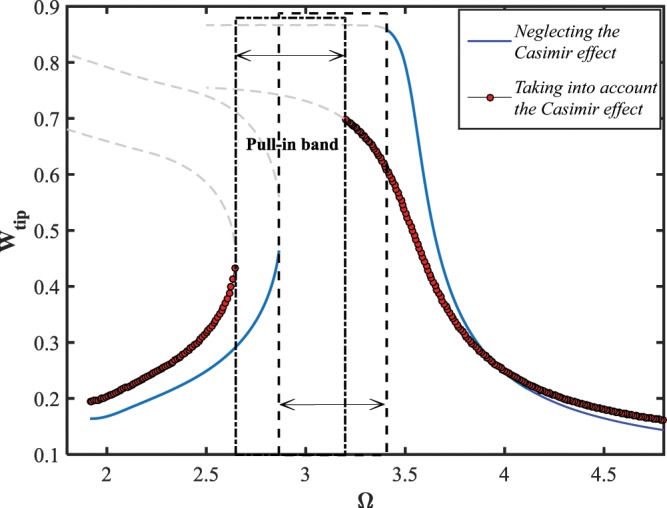
The effect of Casimir regime on the pull-in instability, for ${V}_{DC}=2\,V,\,{V}_{AC}=1\,V\,{\rm{and}}\,{g}_{0}=15\,nm$.

**Figure 14:**
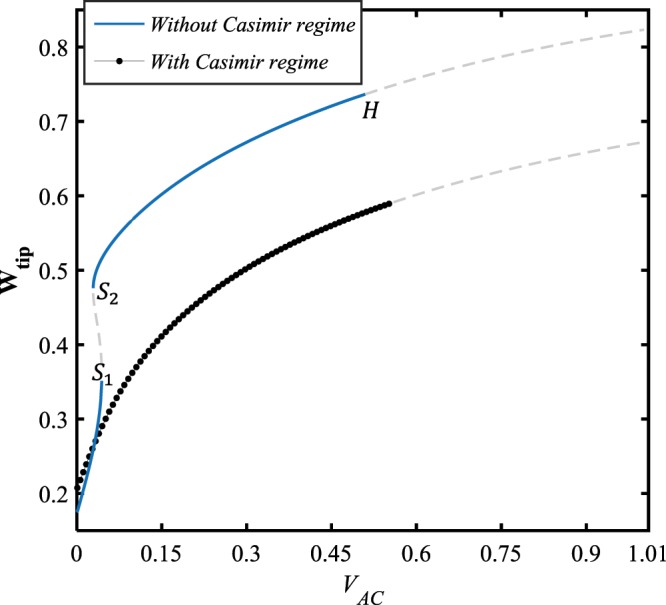
The influence of Casimir force on the force-amplitude behavior of the system, for ${V}_{DC}=3\,V,\,\Omega =3,\,{\rm{and}}\,{g}_{0}=15\,nm$.

In order to investigate the pull-in behavior of the system in the vicinity of the primary resonance, the electrostatic load voltages are set to be ${V}_{DC}=2\,V$ and ${V}_{AC}=1\,V$, Fig. (13). It is shown that, the width of the pull-in band almost remains constant, and only, the unstable region of the excitation frequency shifts to lower frequencies while the effect of Casimir regime is taken into account. Like the previous case, the low-amplitude solution manifolds shift upward, leading to a vast resonance scope as a consequence of this intermolecular interactions. Furthermore, the AC load-amplitude behavior of the NEMS device is indicated in Fig. (14). As seen, the system solution contains three bifurcation points, two saddle-node and one Hopf bifurcation points, while the effect of Casimir force is regraded. However, the low-displacement unstable branch is eliminated and the NEMS dynamic response bifurcates only at one Hopf bifurcation point as the influence of the dispersion force is observed in the model. Moreover, the response level obtained in the presence of the Casimir force is predominantly lower than that computed in the absence of the parameter.

## Conclusive

The impetus of this study was to concern on the large-amplitude resonant motion of a cantilever capacitor made of nanowire. First, the conditions at which the linear and nonlinear models governing the system dynamics result in different outputs were examined. As explained, for NEMS devices with adjustable gap size, the necessary condition which causes the system exhibits large deformation, is that the initial gap value to be large enough in order to provide adequate space for large-amplitude displacement before the pull-in happens. This equals to the case of strong *nonlinear impact factor*. However, the second and sufficient condition which leads to a sharp deviation between the results obtained from the geometrically linear and nonlinear models, is that the electrostatic voltage loads to be categorized in hard excitation class. Under these two simultaneous circumstances, one can conclude that the system response must certainly be captured through a nonlinear dynamics model that contains the effect of geometric nonlinearity.

In the present work, the energy-based approach was utilized to establish the motion’s equation, and the Galerkin decomposition technique employed to describe the system motion through a set of nonlinear ODEs. Also, the influences of other parameters such as size-dependency, surface energy, and dispersion atomic force were included to estimate the system behavior more accurately. The numerical simulation was carried out based on the combination of shooting and arc-length continuation methods to exploit the frequency- and force-response behaviors of the capacitor near the primary resonance.

The research revealed that, the higher the nonlinear impact factor, the less the softening degree of the system response was reported by the nonlinear model. In addition, the amplitude of the wire tip displacement is severely underestimated while the impact of geometric nonlinearity, $\delta$, grows. Furthermore, it was observed that the frequency-response curve shifts to the right almost rigidly, the resonance zone moves to higher frequencies, and the pull-in band width becomes narrower as the small-scale parameter increases. Moreover, it was shown that the system dynamic response is remarkably affected by the surface elasticity parameters. The response level of the NEMS system is highly incremented, and the system periodic orbits are traced along with an intensive softening-type behavior, while the effect of residual surface stress is considered. In addition, this parameter’s effect results in a great wide pull-in band appearing in the frequency-displacement curve, comparing to what is assessed in the absence of the parameter, ${\tau }_{0}$. Unlike the geometric nonlinearity, the influence of the Casimir regime as an intermolecular interaction, on the system solution becomes significant while the split between the two conductors gets small. As further findings, it was seen that the pull-in range is neither widened nor shortened, but only shifts to lower excitation frequencies as the NEMS dynamics is influenced by the Casimir attraction force. Ultimately, it can be expressed that the outputs of this study are useful for cases, in which the NEMS device is provided with a tunable gap and is allowed to experience large displacements.

## Appendix

The components of the nonlinear mass and stiffness matrices, the electrostatic and the Casimir force vectors are obtained as following,

$${M}_{ni}^{1}=\sum _{j}\sum _{k}{q}_{j}{q}_{k}{\int }_{0}^{1}{f}_{ij}(x){\varphi }_{n}{\varphi ^{\prime} }_{k}dx$$

$${M}_{ni}^{2}=\sum _{j}\,\sum _{k}\,{\dot{q}}_{j}{\dot{q}}_{k}{\int }_{0}^{1}\,{f}_{jk}(x){\varphi }_{n}{\varphi }_{i}^{\text{'}}\,dx$$

$${M}_{ni}^{3}=\sum _{j}\,\sum _{k}\,{q}_{j}{q}_{k}{\int }_{0}^{1}\,{g}_{ij}(x){\varphi }_{n}{\varphi }_{k}^{\text{'}\text{'}}dx$$

$${M}_{ni}^{4}=\sum \,_{j}\sum _{k}\,{\dot{q}}_{j}{\dot{q}}_{k}{\int }_{0}^{1}\,{g}_{jk}(x){\varphi }_{n}{\varphi }_{i}^{\text{'}\text{'}}dx$$

$${K}_{ni}^{1}=\sum _{j}\,\sum _{k}\,{q}_{j}{q}_{k}{\int }_{0}^{1}\,({\omega }_{i}^{2}{\varphi }_{i}-{\alpha }_{5}{\varphi }_{i}^{\text{'}\text{'}}){\varphi }_{n}{\varphi }_{j}^{\text{'}}{\varphi }_{k}^{\text{'}}dx$$

$${K}_{ni}^{2}=\sum _{j}\,\sum _{k}\,{q}_{j}{q}_{k}{\int }_{0}^{1}\,{\varphi }_{n}{\varphi }_{i}^{\text{'}\text{'}\text{'}}{\varphi }_{j}^{\text{'}\text{'}}{\varphi }_{k}^{\text{'}}dx$$

$${K}_{ni}^{3}=\sum _{j}\sum _{k}{q}_{j}{q}_{k}{\int }_{0}^{1}{\varphi }_{n}{\varphi }_{i}^{\text{'}\text{'}}{\varphi }_{j}^{\text{'}\text{'}}{\varphi }_{k}^{\text{'}\text{'}}dx$$

$${K}_{ni}^{4}=\sum _{j}\,\sum _{k}\,{q}_{j}{q}_{k}{\int }_{0}^{1}\,{\varphi }_{n}{\varphi }_{i}^{\text{'}\text{'}}{\varphi }_{j}^{\text{'}}{\varphi }_{k}^{\text{'}}dx$$

$${K}_{ni}^{5}=\sum _{j}\sum _{k}{q}_{j}{q}_{k}{\int }_{0}^{1}({\omega }_{i}^{2}{\varphi }_{i}-{\alpha }_{5}{\varphi }_{i}^{\text{'}\text{'}}){\varphi }_{n}{\varphi }_{j}^{\text{'}\text{'}}{\varphi }_{k}^{\text{'}\text{'}}dx$$

$${K}_{ni}^{6}=\sum _{j}\,\sum _{k}\,{q}_{j}{q}_{k}{\int }_{0}^{1}\,({\omega }_{i}^{2}{\varphi }_{i}-{\alpha }_{5}{\varphi }_{i}^{\text{'}\text{'}}){\varphi }_{n}{\varphi }_{j}^{\text{'}\text{'}\text{'}}{\varphi }_{k}^{\text{'}\text{'}}dx$$

$${F}_{i,el}={(1+R\cos (\Omega t))}^{2}{\int }_{0}^{1}\,\frac{{\varphi }_{i}}{(1-\sum {\varphi }_{j}{q}_{j}){{\rm{arccosh}}}^{2}({b}_{0}(1-\sum {\varphi }_{j}{q}_{j}))}dx$$

$${F}_{i,cas}={\int }_{0}^{1}\,\frac{{\varphi }_{i}(1+2\,\mathrm{ln}({b}_{0}(1-\sum {\varphi }_{j}{q}_{j})))}{{(1-\sum {\varphi }_{j}{q}_{j})}^{3}{\mathrm{ln}}^{2}({b}_{0}(1-\sum {\varphi }_{j}{q}_{j}))}dx$$

where,

$${f}_{ij}(x)={\int }_{0}^{x}{\varphi }_{i}^{\text{'}}{\varphi }_{j}^{\text{'}}dx,\,{g}_{ij}(x)={\int }_{1}^{x}\,({\int }_{0}^{x}\,{\varphi }_{i}^{\text{'}}{\varphi }_{j}^{\text{'}}dx)dx$$
